# Imaging Modalities for Early Detection of Pancreatic Cancer: Current State and Future Research Opportunities

**DOI:** 10.3390/cancers14102539

**Published:** 2022-05-21

**Authors:** Katherina P. Farr, Daniel Moses, Koroush S. Haghighi, Phoebe A. Phillips, Claudia M. Hillenbrand, Boon H. Chua

**Affiliations:** 1 School of Clinical Medicine, Faculty of Medicine & Health, UNSW, Sydney, NSW 2052, Australia; kshaghighi@bigpond.com (K.S.H.); boon.chua@health.nsw.gov.au (B.H.C.); 2 Graduate School of Biomedical Engineering, UNSW, Sydney, NSW 2052, Australia; daniel.moses@unsw.edu.au; 3 Department of General Surgery, Prince of Wales Hospital, Sydney, NSW 2052, Australia; 4 Pancreatic Cancer Translational Research Group, School of Clinical Medicine, Lowy Cancer Research Centre, UNSW, Sydney, NSW 2052, Australia; p.phillips@unsw.edu.au; 5 Research Imaging NSW, Division of Research & Enterprise, UNSW, Sydney, NSW 2052, Australia; claudia.hillenbrand@unsw.edu.au; 6 Nelune Comprehensive Cancer Centre, Prince of Wales Hospital, Sydney, NSW 2052, Australia

**Keywords:** pancreatic cancer, pancreatic ductal adenocarcinoma, screening, early detection, pancreatic cystic lesions, MRI, radiomics

## Abstract

**Simple Summary:**

While survival rates for many cancers have improved dramatically over the last 20 years, patients with pancreatic cancer have persistently poor outcomes. The majority of patients with pancreatic cancer are not suitable for potentially curative surgery due to locally advanced or metastatic disease stage at diagnosis. Therefore, early detection would potentially improve survival of pancreatic cancer patients through earlier intervention. Here, we present clinical challenges in the early detection of pancreatic cancer, characterise high risk groups for pancreatic cancer and current screening programs in high-risk individuals. The aim of this scoping review is to investigate the role of both established and novel imaging modalities for early detection of pancreatic cancer. Furthermore, we investigate innovative imaging techniques for early detection of pancreatic cancer, but its widespread application requires further investigation and potentially a combination with other non-invasive biomarkers.

**Abstract:**

Pancreatic cancer, one of the most lethal malignancies, is increasing in incidence. While survival rates for many cancers have improved dramatically over the last 20 years, people with pancreatic cancer have persistently poor outcomes. Potential cure for pancreatic cancer involves surgical resection and adjuvant therapy. However, approximately 85% of patients diagnosed with pancreatic cancer are not suitable for potentially curative therapy due to locally advanced or metastatic disease stage. Because of this stark survival contrast, any improvement in early detection would likely significantly improve survival of patients with pancreatic cancer through earlier intervention. This comprehensive scoping review describes the current evidence on groups at high risk for developing pancreatic cancer, including individuals with inherited predisposition, pancreatic cystic lesions, diabetes, and pancreatitis. We review the current roles of imaging modalities focusing on early detection of pancreatic cancer. Additionally, we propose the use of advanced imaging modalities to identify early, potentially curable pancreatic cancer in high-risk cohorts. We discuss innovative imaging techniques for early detection of pancreatic cancer, but its widespread application requires further investigation and potentially a combination with other non-invasive biomarkers.

## 1. Introduction

Pancreatic ductal adenocarcinoma (PDAC) is estimated to be the second leading cause of cancer-related deaths by 2030 [1]. The high mortality rate for this disease is partly due to late presentation rendering therapeutics ineffective [2]. Ninety percent of PDACs are sporadic in origin; around 10% of cases occur in hereditary and familial predisposition syndromes [3]. To date, certain risk factors such as smoking, alcohol use, and chronic pancreatitis are found to be strongly associated with PDAC. The risk for PDAC increases with age; more than 80% of cases occur between ages 60 and 80 years [3]. The mean size of PDAC is approximately 3.1 cm, and approximately 80% of patients manifest distant metastases or locally advanced disease at presentation, which make them ineligible for surgical intervention. Since most patients are diagnosed at advanced stages due to the lack of specific symptoms, and the prognosis is linked to the stage of disease at diagnosis, there is a need for robust early detection methods. Although the goal of early detection in PDAC remains laudable, the screening for PDAC in the general population of asymptomatic individuals is not recommended [4]. However, certain high-risk individuals may derive benefit from screening and surveillance, facilitating earlier diagnosis and life-saving surgical intervention, which remains the most effective curative modality [5]. The most suitable imaging modalities for early detection of PDAC have yet to be identified. We reviewed current knowledge on imaging modalities used for early detection of PDAC. Factors associated with a high risk of PDAC development would also be discussed. Finally, we would review the emerging roles of novel imaging modalities in PDAC detection.

For this narrative review, our search strategy consisted of a general search of diagnostic and therapeutic images in pancreatic cancer, followed by a search of specific imaging modalities and, finally, reviewing the papers for leads to other—not yet included—imaging techniques.

## 2. High-Risk Groups Relevant for PDAC Early Detection

Patients with cystic lesions are at increased risk for developing PDAC [6]. There are several risk factors for developing a cystic precursor lesion and associated PDAC. The relationship of diabetes mellitus and PDAC is an intense area of research interest, with significant progress being made in interactions of diabetes and PDAC development [7]. Further, patients with chronic pancreatitis are at an increased risk of developing a cystic precursor lesion and associated PDAC [8]. Certain genetic syndromes and a familial PDAC have been shown to pose a risk [9]. Based on the literature, we focus on patients with pancreatic cystic lesions, high-risk individuals with a familial PDAC risk, at risk cohorts with pancreatitis, genetic syndromes and germline mutations, and elderly patients with new-onset diabetes (Table 1).

### 2.1. Familial PDAC

A family history of pancreatic cancer is observed in 5% to 10% of patients with PDAC. Gene mutations in CDKN2A (p16), BRCA2, and PALB2 are associated with PDAC [10,11,12,13]. Familial PDAC is defined as having two or more first-degree relatives with PDAC. The relative risk for PDAC is 2.41 in sporadic cases (i.e., families with only one relative with PDAC or with multiple PDACs in more distant relatives and/or spouses with PDAC), whereas the risk increases to 6.79 and 17.2 times in cases with two and more first-degree relatives with PDAC, respectively [14]. In the familial PDAC kindreds, risk varied by the number of first-degree relatives with PDAC, such that risk was higher in individuals with three first-degree relatives who had PDAC (Standardized Incidence Ratio, SIR = 17.02; 95% CI = 7.34 to 33.5; *p* < 0.01), but lower in individuals who had two first-degree relatives with PDAC (SIR = 3.97, 95%CI = 1.59 to 8.2, *p* = 0.05) or with one affected first-degree relative (SIR = 6.86, 95% CI = 3.75 to 11.04, *p* < 0.001) [15]. Whereas risk was higher for familial PDAC kindred members who had one first-degree relative with PDACs compared with two, the confidence intervals for these two estimates largely overlap. Moreover, a higher risk of PDAC has been observed among familial pancreatic cancer kindreds with younger-onset PDAC (age, <50 years; standardized incidence ratio = 9.3%) [16]. A familial history of pancreatic cancer suggests a high risk for PDAC, and the incidence of PDAC depends on the number of first-degree relative with PDAC. Identification of a family history of PDAC is therefore, important.

### 2.2. Inherited Cancer Predisposition Syndromes

Several cancer predisposition syndromes are known to increase PDAC risk (Table 1) [17,18]. Although genetic defects likely remain unknown, several genetic syndromes associated with PDAC have been discovered. The inherited cancer syndromes, such as hereditary breast-ovarian cancer syndrome, hereditary pancreatitis, Peutz–Jeghers syndrome, familial atypical multiple mole myeloma, Lynch syndrome, Li–Fraumeni syndrome, familial breast-ovarian cancer with, and hereditary nonpolyposis colorectal cancer have all been associated with increased risk of developing PDAC [19,20,21,22,23]. However, because of their rarity, they in total account for only a small fraction of PDACs.

### 2.3. Pancreatic Cystic Lesions

**Figure 1:**
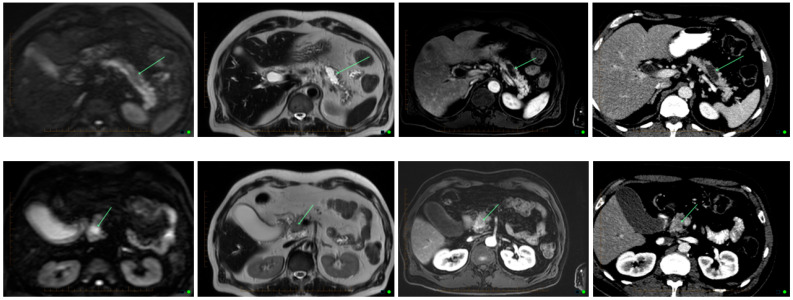
Transformation in IPNM on MRI and CT. From left to right- Diffusion weighted imaging, T2, post contrast T1 and CT images in the same patient. Top row shows a simple cystic lesion consistent with a side branch IPMN, showing no malignant features. Bottom row shows a cystic lesion displaying solid enhancing components with restricted diffusion, consistent with malignant degeneration within an IMPN. Arrows indicate cystic component with no cancer (top), and with cancer (bottom).

Pancreatic cystic lesions can be divided into three main types: inflammatory, serous and mucinous, as well as other rare cyst types. For the scope of this review, mucinous cystic lesions are described as precursor lesions, harbouring a greater potential for malignancy. It is now well-known that most PDAC originate from microscopic pancreatic intraepithelial neoplasia (PanIN) and macroscopic precursor lesions. PanIN refers to microscopic, intraductal neoplasms (by definition <5 mm) lined by gastric–foveolar epithelia of varying degrees of architectural and cytologic atypia. These cannot be detected by current imaging modalities. While PanINs contribute to most of PDACs, a significant proportion of PDACs arise from macroscopic mucinous neoplasms, such as intraductal papillary mucinous neoplasms (IPMN) and mucinous cystic neoplasms (MCN) (Figure 1). These cystic precursor lesions share many genetic alterations found in PDACs. Up to 15% of PDACs are thought to arise from mucinous pancreatic cysts, which include IPMNs and MCN [24]. The incidence of PDAC was reported to be 2% in 349 patients with IPMN who were observed for 3.7 years [25]. Another study reported that PDAC occurred in 5 of 60 (8%) patients with IPMN (with a diameter <10 mm), who were observed for 87 months, and the 1- and 5-year mortality rates were 1.1% and 6.9%, respectively [26]. The presence of any pancreatic cyst, including IPMN, was reported to be a high-risk factor for PDAC development, accounting for 0.95% of patients with pancreatic cysts per 1 year, which was 22.5 times higher than that in individuals without pancreatic cystic lesions [27]. Patients harbouring a cystic lesion are more likely to progress to cancer than even those with family history of PDAC making them the prime target population for screening and surveillance modalities [16]. However, the matter is complicated by cystic lesions presenting a variable risk of malignant transformation (Figure 1).

**Figure 2:**
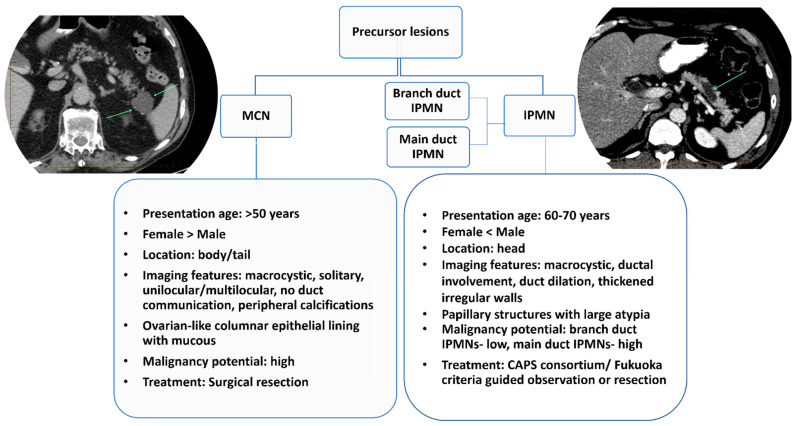
Characteristics of pancreatic cystic lesion types and their key differences. IPMN= intraductal papillary mucinous neoplasms; MCN= mucinous cystic neoplasms.

IPMNs are macroscopic (>1 cm by definition) cystic tumours characterised by intraductal growth of papillary lesions with typically copious and thick mucin production. IPMNs commonly originate from the main pancreatic duct, its contributing branches, or possible mixed origin. They are more common in elderly men (>65 years). Most patients with IPMNs are asymptomatic; some patients may have nonspecific symptoms of abdominal pain, jaundice, as well as symptoms due to exocrine and/or endocrine pancreatic insufficiency. The pathologic nomenclature of IPMNs is complex and evolving. The incidence and type of invasive malignancy developing in IPMNs differ from one histologic type to another. Pancreatic ductal communication is the key feature differentiating these from other cystic neoplasms. Main duct IPMN is characterized by cystic dilatation of the main duct, variable ductal wall thickening, and thick, abundant mucin that distends and obstructs the duct (Figure 2). Main duct IPMN have a higher predisposition for malignant transformation, compared with branch duct IPMNs. A longitudinal study by Levy et al. demonstrated the 5-year actuarial risk of progression to high-grade dysplasia among main duct IPMNs was 63%, while 15% in the branch duct IPMNs [25]. Patients with an IPMN are at an increased risk for developing not only IPMN-associated PDAC but also PDAC independent from the IPMN, concomitant carcinomas [24]. Existing guidelines recommend immediate surgical resection for any lesion with high-risk stigmata and regular surveillance with interval imaging for all mucinous or indeterminate lesions [28]. Surveillance protocols vary between the different guidelines [29].

Mucinous cystic neoplasms, the least common macroscopic precursor lesion, differ from IPMNs in that they do not communicate with the ductal system, and they are almost always solitary. In addition, 98% of mucinous cystic neoplasms occur in perimenopausal women with a distinct proclivity to involve the distal pancreas (>90% occur in the tail region) and histologically characterised by a pathognomonic spindle cell ovarian-type stroma. Surgical resection is recommended in mucinous cystic neoplasms of greater than 4 cm, symptomatic tumours, and enhancing mural nodules. European evidence-based guidelines suggest that asymptomatic or smaller mucinous cystic neoplasms without suspicious features need long-term surveillance (every 6 months for 1st year and then annual) as long as they do not have surgical contraindications [30]. The risk of malignant transformation of mucinous cystic neoplasms is lower than that of IPMN; approximately 16% of mucinous cystic neoplasms are associated with invasive malignancy. When malignancy develops, it is typically of the tubular carcinoma variant of PDAC, but lymph node metastasis is usually not seen.

### 2.4. Newly Onset Diabetes Mellitus

Although the association between diabetes mellitus and PDAC has been known since 1800s, the intricate relationship between the two conditions has yet to be fully understood. Diabetes appears to have a multidirectional association with PDAC. The onset of diabetes has been shown to precede occurrence of PDAC by a few years and resolve after the resection [31]. The mechanism can be attributed to paraneoplastic phenomenon, leading to induced insulin resistance from pancreatic polypeptide deficiency. Although long standing type 2 diabetes is a modest risk factor (1.5–2-fold increased risk) for PDAC, newly onset diabetes may be a manifestation of PDAC [32]. It has been suggested that up to 85% of pancreatic cancer patients have diabetes or hyperglycaemia which can manifest 2–3 years before the development of PDAC [32]. This has been attributed to growth stimulation by endogenous hyperinsulinemia. Increased risks due to obesity and metabolic syndrome are also thought to arise from elevated insulin levels. For this newly identified high-risk group, there are no established guidelines or screening programs. Increasing epidemiological, clinical, and experimental evidence that newly onset diabetes is a clinical manifestation of asymptomatic PDAC provides hope for the early detection of PDAC in patients with diabetes.

### 2.5. Pancreatitis

Acute pancreatitis can be the initial clinical presentation of PDAC and can precede the diagnosis of PDAC by several weeks or months [33]. Few studies have reported a detailed list of the aetiology of acute pancreatitis in their study cohort [34,35,36,37]. Estimated 1.7–3.6% of patients with acute pancreatitis were finally diagnosed with PDAC. The pooled average was 2.03% in 2945 patients in these studies [33]. Duell et al. reported a nearly seven-fold increased relative risk for pancreatic cancer in individuals with a history of pancreatitis (adjusted odds ratio, 6.9; 95% CI, 3.4–14.1) [38]. Another study reported that 1.45% of patients with acute pancreatitis developed PDAC within the 2-year period, and the incidence of PDAC reduced in the third year; further, age 40 years and older was an added risk factor for PDAC [39]. Cases of carcinoma in situ were recently reported to be the cause of acute pancreatitis [40]. Acute pancreatitis can be an indicator of PDAC, and patients with acute pancreatitis should be observed for 2 years using diagnostic imaging techniques [33]. In chronic pancreatitis, >2-year observation showed that the relative risk for PDAC was 16.5 to 26.7, and the incidence ratios of PDAC occurrence were reported to be 1.1%, 1.8%, and 4.0% in 5-, 10- and 20-year observations, respectively [41]. Hereditary pancreatitis is defined by the following: acute recurrent pancreatitis or chronic pancreatitis in two and more members of a family; an absence of a history of alcohol abuse in at least one patient; and pancreatitis in at least one brother or sister younger than 40 years. If the patient has a p.R122H or p.N291 mutation on PRSS1, the diagnosis of hereditary pancreatitis is confirmed, irrespective of the definition. In hereditary pancreatitis, cumulative lifetime risk of PDAC is 40% [42]. The proportions of patients developing PDAC due to hereditary pancreatitis are 10%, 18.7%, and 53.5% in 50-, 60-, and 75-year-old patients, respectively [42]. Familial history of pancreatitis should be considered when examining a patient with pancreatitis.

## 3. Screening for PDAC

To date, there is no evidence that screening for PDAC or treatment of screen detected PDAC improve disease-specific morbidity or mortality [4]. However, certain high-risk individuals with greater than 5% lifetime risk of PDAC, or a five-fold increased relative risk, may derive benefit from surveillance (Table 2). Several large academic centres have conducted screening programs for these asymptomatic high-risk individuals, based on genetic predisposition [43,44,45]. Preliminary evidence of benefit from pancreatic cancer surveillance in international screening protocols is encouraging. Depending on the age and other characteristics of the study population and the imaging modalities, the prevalence of precursor lesions identified by screening has ranged from 6–52% [43]. Since nearly all patients with symptomatic invasive PDAC and many of those with asymptomatic PDAC diagnosed in screening programs die of their malignancy, the literature suggests that the goal of a pancreatic cancer screening and surveillance program should be to detect and selectively treat asymptomatic non-invasive high-grade precursor neoplasms, rather than focusing screening efforts to detect invasive cancers [27].

### Current Guidelines for Screening Programs

The International Cancer of the Pancreas Screening (CAPS) Consortium met in 2020 to update the consensus recommendations for the management of individuals with increased risk of PDAC based on family history or germline mutation status [9]. An international consortium of experts recommended pancreatic screening and surveillance be evaluated with an estimated lifetime risk of PDAC of >5%. The main goal of surveillance was to identify high-grade dysplastic precursor lesions and T1N0M0 pancreatic cancer. CAPS experts agreed that for those with familial risk, surveillance should start at age 50, or 10 years earlier than the youngest relative with pancreatic cancer. CAPS recommended surveillance tests were endoscopic ultrasound and MRI/magnetic retrograde cholangiopancreatography (MRCP) (Table 2). Annual surveillance was recommended in the absence of concerning lesions. Multiple institutions within the CAPS consortium are currently generating data on pancreatic cancer surveillance protocols in high-risk individuals to determine the clinical benefits of early detection of PDAC and long-term outcomes [46]. However, there is currently no proven long-term benefit of pancreatic cancer surveillance and as the actual number of patients diagnosed with PDAC in these studies is small, pooling of data from individual screening trials is needed to accumulate sufficient evidence of a clinical benefit. CAPS main areas of disagreement included if and how surveillance should be performed for hereditary pancreatitis, and the management of indeterminate lesions [9]. In that, the decades-old challenge remains to identify the high-risk patients harbouring early malignancy or precursor lesions, thereby accurately determining the necessity of surgery.

## 4. Traditional Imaging Modalities for Pancreatic Cancer Detection

The clinical indications for diagnostic imaging of PDAC include diagnosis of the primary tumour, resectability assessment, evaluation of distant metastasis, and evaluation of treatment response. In the context of early detection, imaging strategies can be grouped into traditional and novel applications.

Currently, the most common modalities to image the pancreas include CT, MRI, and endoscopic ultrasonography (EUS); however, access to these modalities is limited to diagnosis and staging. Over the past two decades, multiple studies have evaluated the accuracy of EUS, CT and MRI for detection of primary tumour in the pancreas, including their value in the context of early detection in high-risk cohorts (Table 3) [31,45,47,48]. Even though EUS has excellent performance with visualizing and diagnosing PDAC, it is mainly used as part of the workup to obtain fine needle aspiration or biopsy material in patients suspected of having a primary tumour. The reason is that EUS is not a readily accessible imaging modality and is highly dependent on operator skills. Being an invasive procedure, the risks of EUS include pancreatitis, procedural pain, puncture and perforation, and risks of anaesthesia [49,50,51]. Even though incorporation of advanced imaging techniques with EUS is ongoing emerging area, operator dependencies and procedural risks remain challenges for this modality. The consensus opinion is that a pancreatic CT scan is warranted for evaluation of a suspected PDAC. Even though recent advancements in CT technology may lead to an increased detection of small pancreatic tumours, MRI was reported to have a greater ability to detect pancreatic lesions than CT in a recent comparison study [27]. A recent systemic review and meta-analysis on modern imaging modalities available for the diagnostic of PDAC, established MRI to have superior sensitivity, specificity, and diagnostic accuracy, as compared to CT, and EUS [52]. Overall, proportional meta-analysis of available data showed that MRI has a sensitivity, specificity and diagnostic accuracy of 93% (95% CI = 88–96), 89% (95% CI = 82–94) and 90% (95% CI = 86–94) respectively for the detection of PDAC.

In the recent review, Arnone et al. investigated the role of 18F-fluorodeoxyglucose (FDG) positron emission tomography (PET) in PDAC, as its role is currently not clear and considered to be “under development” [53]. In the literature, the clinical impact of FDG PET in PDAC in different disease phases, such as diagnosis, preoperative staging, prognosis, tumour recurrence and treatment response, is investigated [53,54,55,56]. Although the routine use of FDG PET is not well established, functional imaging can provide useful information and hold a relevant position in the whole management of PDAC. Besides conventional anatomical imaging, such as CT and MRI, molecular imaging with FDG PET can be used in all phases of disease but, considering the limited role at diagnosis for a low specificity and for limited results about the use in response to therapy assessment, PET showed the potential best performances for preoperative staging, recurrence detection, and prognosis estimation of PDAC [53,54,55].

MRI and MRCP are superior to CT in the assessment of ductal communication of the cystic lesions as well as depiction of internal characteristics such as septations and mural nodules, although both modalities are equivalent in early detection and characterisation. Moreover, MRI is preferable to CT for imaging surveillance of cystic lesions, particularly in young patients because of cumulative deleterious effects of ionizing radiation with CT. Thickened irregular septae, enhancing mural nodule, or solid components within the IPMN are suspicious for malignancy (Figure 1). Specific, worrisome imaging features that strongly suggest malignancy include main duct diameter of greater than 1 cm, enhancing mural nodule greater than 5 mm, cystic growth rate of greater than 5 mm per year, and cyst greater than 4 cm in diameter [6]. IPMNs are multicentric in 20–40% of cases, which emphasises the importance of following-up patients after surgical resection. Mucinous cystic neoplasms commonly appear as large, complex cystic masses with variable CT density due to haemorrhage, necrosis, and calcifications (Figure 2). MRI and MRCP exquisitely depict the internal septations, variable fluid signal intensity, and solid components of multilocular cystic masses. As with IPMNs, the malignant mucinous cystic neoplasms show heterogeneity, thickened septations, and enhancing solid components. Imaging modalities in the context of solid lesions, and their advantages and disadvantages for the purpose of early detection are presented in Table 3.

The limitation of current imaging-based screens is that upon identification of potentially benign lesions, subsequent invasive evaluation by collecting tissue biopsies is still required to confirm diagnosis. Early diagnostics to identify carcinomas in situ are often challenging due to the detection limit of current radiological methods and how chronic pancreatitis shows similar fibrotic features to pancreatic cancer. Furthermore, morphologic tumour changes significant on imaging, appear much later than functional and metabolic changes. Thus, a considerable concern is that current imaging methods might not be adequate to identify tumours in the pancreas at an earlier stage when treatment would be optimal. A retrospective review of CT scans carried out for other indications showed no evidence of a pancreatic mass in most patients 6 months or earlier before the diagnosis of PDAC [57]. Thus, there is a need for advanced imaging techniques to improve detection at an earlier stage than is presently possible. More importantly, each of these imaging modalities have variable sensitivities (Table 3). Early detection requires the ability to detect T1N0M0 PDACs with fewer cancer cells or high-grade dysplastic premalignant lesions. Current imaging techniques are limited in their ability to detect PDAC at an earlier stage. Furthermore, current image-based guidelines are inadequate to distinguish benign from malignant lesions. There continues to be a need for accurate imaging and molecular biomarkers capable to identify and predict the malignant potential of cystic lesions to enable risk stratification and effective intervention. Therefore, there is a dire need to improve imaging accuracy and identify specific imaging features for early-stage detection.

## 5. Novel Uses and Techniques for Imaging of PDAC

Among the novel imaging modalities available for PDAC detection are functional imaging techniques.

### 5.1. Diffusion Weighted Imaging

Diffusion weighted imaging (DWI) is a relatively new MRI technique that reflects changes in water mobility caused by interactions with cell membranes and macromolecules, and alterations in the tissue microenvironment. Therefore, DWI provides a tissue contrast that is different from that of conventional T1- and T2-weighted MRI images. In as much as DWI offers quantitative measurements of the diffusivity of water described by the apparent diffusion coefficient (ADC), it also represents microcirculation of blood flow. Therefore, in pancreatic cancer, ADC values are usually lower than in normal pancreatic tissue. Using DWI, Kamisawa et al., evaluated its clinical utility in patients with cancer and autoimmune pancreatitis, and assessed whether DWI could help differentiate cancer from pancreatitis [58]. They determined that ADC values were significantly lower in pancreatitis than in PDAC and normal pancreas, underscoring the potential of DWI as a diagnostic test [59]. DWI offers quantitative measurements of blood perfusion and the molecular diffusion of water. DWI has proven helpful for the identification of subtle lesions with diffusion restriction and a preferred modality for assessing cystic lesions in the pancreas [60,61]. Previous studies reported high diagnostic performance for identification of pancreatic ductal adenocarcinoma using DWI, with reported accuracy, sensitivity, and specificity of 96%, 96%, and 99%, respectively [62]. Similarly, Kartalis et al. found very high diagnostic performance of DWI (92% sensitivity, 97% specificity, 96% accuracy) to differentiate malignant from benign pancreatic lesions [63]. Additionally, several studies demonstrated that DWI is a reliable tool to identify liver metastases from pancreatic tumours and to predict the aggressiveness of such lesions, as ADC has shown to be lower in patients with worse clinical course and prognosis [64,65].

ADC values reflect both molecular diffusion and microcirculation of blood (perfusion). The Intravoxel incoherent motion (IVIM) approach can separate signal attenuating effects of microcirculation (“pseudo-diffusion”) from molecular diffusion and thereby provide additional information to characterise focal pancreatic lesions, verifying more restricted diffusion in solid malignant tumours versus benign inflammatory ones [66]. IVIM-based perfusion MRI, which does not require contrast agents, is gaining momentum, especially for oncologic applications. Perfusion imaging, such as IVIM MRI, is an important diagnostic imaging modality to evaluate neoangiogenesis or microvasculature heterogeneity [67]. Until now, however, there have been only a few studies in which the value of IVIM was explored to differentiate malignant pancreatic tumours from benign lesions [67,68]. Technological improvements have made possible the routine use of DWI during abdominal MRI study. Several authors have reported that the addition of the DWI sequence can be of value for the evaluation of patients with PDAC, especially improving the staging. Nevertheless, it is still unclear whether and how DWI could be helpful for identification, characterization, prognostic stratification, and follow-up during treatment [64]. Furthermore, there are several technical difficulties in applying DWI to the pancreas, including respiratory motion, and field inhomogeneity due to gas in the surrounding stomach and intestines [62,68].

### 5.2. Dynamic Contrast Enhanced MRI

MRI can also provide other advanced techniques such as dynamic contrast enhanced MRI (DCE-MRI) for evaluation of perfusion. Potential major interest of functional imaging is to show early fibrotic and metabolic changes in pancreatic parenchyma, despite the absence of morphological changes. Granata et al. evaluated functional MRI to differentiate pancreatic tumours, peritumoural inflammatory tissue, and normal pancreatic parenchyma by means of DCE-MRI, diffusion kurtosis imaging, and IVIM DWI-derived parameters [66]. Further, several studies evaluated the feasibility of DCE-MRI for the characterization of solid pancreatic diseases. Having produced some promising results, DCE-MRI accuracy in the evaluation of pancreatic cancer remains unclear. Tumour hypoxia is a significant factor in cancer progression, angiogenesis, metastasis, and resistance to therapy [69]. It has additionally been identified as a marker of degree of fibrosis and poor vascularisation [70]. The potential of DCE-MRI to assess the extent of hypoxia in tumours has been investigated in several studies [71,72]. Together, the studies imply that DCE-MRI can provide valuable information on targeting the hypoxic status of PDAC.

### 5.3. Hyperpolarised MRI

Hyperpolarised MRI can identify metabolic aberrations in the pancreas that indicate preneoplasia. Metabolic MRI imaging with hyperpolarised agents enables detection and monitoring of the progression of precursor lesions towards invasive PDAC. In that, hyperpolarised MRI can identify metabolic aberrations in the pancreas that indicate preneoplasia [73]. Recently the hyperpolarisation of compounds enriched with ^13^C pyruvate has been demonstrated in animal models and now in preliminary clinical studies [73]. Metabolic MRI imaging with hyperpolarised [1-^13^C] pyruvate enables detection and monitoring of the progression of precursor lesions towards invasive PDAC [74].

Hyperpolarised [1-^13^C] MRI has been proven feasible in experimental models, differentiating exocrine pancreas, pancreatitis, and pancreatic cancer tissue by the enzymatic conversion of pyruvate-to-lactate and pyruvate-to-alanine (represented by the alanine-to-lactate ratio or alanine transferase/lactate dehydrogenase ratio), and demonstrated that this relationship correlates with disease progression and treatment response [73]. Clinical data on hyperpolarised MRI for characterization of heterogeneous and hypoxic pancreatic tumours in two patients with PDAC have been published. Hyperpolarised [1-^13^C] MRI successfully differentiated pancreatic tumour tissue from surrounding tissue >30 s after the injection via [1-^13^C]lactate and [1-^13^C]alanine production [75].

### 5.4. MR Elastography

MR elastography (MRE), is another potential method to detect fibrosis. MRE, a multifrequency magnetic resonance elastography technique with noise-robust data post processing, has been introduced to the field of cancer imaging recently [76,77]. It provides high-resolution parametric maps, quantifying tissue stiffness and fluidity. While stiffness is well known as the property assessed by palpation, fluidity is relatively new to tumour characterization. MRE showed promising results to allow differentiation of PDAC and pancreatitis features with high accuracy [78]. In prospective clinical trial, Zhu et al. evaluated the diagnostic performance of MRE in distinguishing between PDAC and autoimmune pancreatitis. Incorporation of elastography (stiffness measurements) in the characterisation of solid pancreatic lesions resulted in higher detection rates. The results showed that both stiffness and fluidity allowed distinguishing PDAC from autoimmune pancreatitis, with AUCs of 0.906 for stiffness, 0.872 for fluidity, and 0.842 for conventional MRI [78].

Functional MRI techniques, therefore, have shown a potential to play an important role in earlier tumour detection of PDAC.

### 5.5. Multidetector CT

There is increasing evidence that dual energy contrast enhanced CT is superior to monoenergy CT [79,80]. Dual energy scans can simultaneously image the patient with 2 energies of X-rays. The contrast-to-noise ratio between pancreatic cancer and normal parenchyma can be improved using dual energy technique. Clinically, this results in maximizing the contrast to typically poorly vascularized pancreatic cancers [81,82]. This offers potential to increase the detection of small or otherwise isoattenuating pancreatic tumours. Further prospective evaluation of dual-energy CT in appropriate populations may be warranted.

### 5.6. Nanomaterials and Molecular Imaging for Advancing Pancreatic Cancer Imaging

Molecular imaging has emerged as a potential way to identify smaller lesions, translating into the potential to diagnose at a much earlier stage than is available. Molecular imaging has the benefit of being able to identify differences between tumour and normal tissue on a molecular level, not based on morphological differences. Being able to combine molecular imaging with conventional imaging in, for example, molecular ultrasound, fluorescence endoscopy and PET/MRI, could have important implications for patient outcomes. Advanced molecular imaging has come to play an integral role in the management of gastro–entero–pancreatic neuroendocrine neoplasms. Somatostatin receptor targeting PET/MRI with liver-specific contrast agent has shown a strong potential for multiparametric evaluation of neuroendocrine neoplasms [83]. So far, the following targets have been investigated- imaging tumour vasculature, tumour epithelial cells, plectin 1, receptor tyrosine kinase axl, bombesin receptors and MUC4 MRI approach [84]. In addition to standard imaging techniques, experimental imaging strategies, such as those utilising molecular probes, nanoparticle-based agents, and tagged antibodies are actively being explored experimentally [85].

Nanotechnology has a potential to non-invasively differentiate between tumour and stromal elements in pancreatic cancer, thus nanoparticles could be used to target tumour elements and stromal elements of pancreatic cancer. Nanotechnology is defined as the manipulation of organic or inorganic materials to form structures on the scale of nanometres. Recently, advances in nanotechnology have provided great opportunities for strategies in advancing cancer diagnostics, imaging, and therapeutic drug delivery [86]. Nanoparticles have the potential to increase the efficacy per dose of a therapeutic or imaging contrast formulation by increasing its bioavailability. These nanoparticles have the potential to enhance the contrast between the delayed uptake (hypoperfusion) of the hypovascular tumours when compared to the normal parenchyma during the arterial and venous phase with conventional radiology approaches. In addition, multifunctional nanoparticles or hybrid systems have also shown great promise. These nanomaterials possess greater signal amplification further improving the diagnostics and imaging sensitivity, while also having the capacity to be used as a therapeutic [87,88]. Challenges in nanoparticles imaging agents clinical application is time-dependent biodistribution and subsequently organ-specific accumulations [89]. Methods to address key technological challenges of nanoparticles, such as scaled-up synthesis and performance optimization, will be essential in ensuring the clinical success of future nanoparticle formulations. A number of studies highlight the potential for the combined use of molecular markers which target pancreatic cancer cells, the surrounding tumour stroma and nanotechnology to improve the specificity and sensitivity of current pancreatic cancer imaging modalities [90,91,92,93]. Extracting quantitative imaging features of pancreatic parenchyma that indicate risk, can therefore inform screening strategies.

### 5.7. Radiomics and Artificial Intelligence-Assisted Methods

The quantitative analysis of medical images data and the extraction of imaging features, also called ‘radiomics’, represent an emerging approach in personalized medicine and advanced diagnostics, especially for disease characterization or outcome prediction. The interest towards radiomics is rapidly growing in the multidisciplinary cancer community as it shows an interesting pertinency and efficacy to answer several clinical questions arising in the management of patients affected by PDAC. In the recent systematic review by Casa et al. radiomics analysis showed to be a promising approach to evaluate PDAC from diagnosis to treatment response prediction [94]. Despite several limitations (variability of acquisition protocols, lack of technical standardization), radiomics could potentially have an important role in providing reliable risk stratification, facilitating surgical choices, predicting clinical response after treatments, allowing differential diagnosis between cancer and other benign pancreatic abnormalities and predicting histological examination, disease differentiation grade or specific gene mutations [94]. DWI radiomics for texture and shape feature evaluation, combined with machine learning methods, have been increasingly applied to investigate DWI’s diagnostic and prognostic roles in several tumours [62]. This evaluation is based on the automated extraction of a number of image features beyond human perception, which can be “seen” only by computers. This line of research will probably represent the future direction for providing an objective evaluation of medical images. Imaging represents more sensitive and specific information for parenchyma and duct of the pancreas than personal health data. Radiomic analysis of medical images using for example, deep learning might allow identification of unique features in pre-diagnostic images to allow accurate prediction of PDAC in the near future.

## 6. Summary and Strategies for the Future

While exact benefit of PDAC screening remains unclear, screening of the general population is not recommended due to the low disease incidence and high costs. The main goal, therefore, is early detection of asymptomatic high-grade precursor lesions and non-invasive PDAC through targeted screening of high-risk populations to enable detection of resectable lesions. In effect, the task remains to identify the most at risk within this high-risk population. Due to relatively low incidence of PDAC, pooling of data from individual screening trials is needed to accumulate sufficient evidence of a clinical benefit. There has been recently significant improvement in pancreatic imaging using the multi-modality approach. Rapidly developing novel imaging techniques are expected to become widely used once their role in early detection of pancreatic cancer is established. Improved understanding of cancer precursors will shed light on the importance of early detection of such lesions, particularly in high-risk patients. The key to early detection is identifying high risk individuals, for whom imaging will be relevant, and using established imaging modalities with novel techniques in the setting of multi-modality approach. Finally, nanotechnology will have an important role in realising the goal for early detection and diagnostics of PDAC. Taken together, the future strategy may be the formation of multi-parametric risk models that combine imaging and clinical data, whereas artificial intelligence applied to imaging offers the possibility to detect early-stage cancer and thus extend survival for patients with PDAC.

## Figures and Tables

**Table 1:** High risk criteria for PDAC.

Criteria	Feature
CT findings	Pancreatic cystic lesion >2 cm; presence of pancreatitis; pancreatic duct dilation >6 mm; duct stricture, IPMN
Diabetes	Newly onset diabetes (<36 m) or worsening of established diabetes/hyperglycaemia
Pancreatitis	Chronic pancreatitis, Hereditary Pancreatitis
Biomarker	Elevated serum CA 19-9
Familial PDAC	More than one blood relative with PDAC; at least one first-degree relative with PDAC; PDAC before 50, other family history
Genetic syndromes	Peutz–Jeghers Syndrome (STK11 mutation); Hereditary pancreatitis (PRSS1 and SPINK1 genes mutation); Lynch Syndrome (MMR mutation); Li–Fraumeni Syndrome (p53 mutation), Familial Atypical Multiple Mole Melanoma (CDKN2A gene mutation)
Germline mutations	BRCA 1, BRCA 2 mutations; ATM mutation; PALB2 mutation

PDAC = pancreatic ductal adenocarcinoma; IPMN = intraductal papillary mucinous neoplasms.

**Table 2:** Existing screening programs for pancreatic cancer in high-risk individuals.

	Who? Targeted Population	How? Screening Program
Inherited PDAC (10%)	Individuals with familial pancreatic cancer (at least one pair of first-degree relatives), inherited pancreatic cancer syndromes	Annual endoscopic ultrasound or MRI
Non-inherited PDAC (90%)	Individuals with cystic tumours of the pancreas (IPMNs or MCNs)	Endoscopic ultrasound or MRI 6–24 months (if worrisome features present)
	Individuals with other predispositions	No established screening
	Individuals with symptoms	Refer to multi-disciplinary diagnostic centres

PDAC = pancreatic ductal adenocarcinoma; MRI = magnetic resonance imaging; IPMN = intraductal papillary mucinous neoplasms; MCN = mucinous cystic neoplasms.

**Table 3:** Performance of imaging modalities for solid PDAC lesions for early detection.

	Advantages	Disadvantages
Computed tomography (CT) 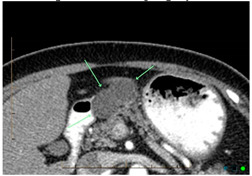	High sensitivity and specificity (76–92% and 67% respectively) Standardized available protocol- pancreatic protocol CT Multidetector CT Good spatial and temporal resolution Lower cost and greater availability	Radiation exposure with the risk of secondary cancer attributable to the CT procedure Performance depends on ability to administer intravenous Iodine contrast Allergies to CT contrast agents (common) Cannot detect iso-attenuating PDACs with indistinct borders and small pancreatic tumours
Endoscopic ultrasound (EUS) 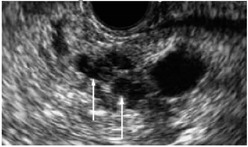	High sensitivity and specificity (72% and 90% respectively) Excellent resolution for small lesions Mainly used as part of the work-up to obtain biopsy (FNA) for tissue diagnosis	Performance varies by disease T stage Invasive procedure, not practical for routine follow-up Not readily accessible imaging modality Highly dependent on technical skill of the operator Limitations for evaluating solid pancreatic lesions Procedural risks
Magnetic resonance imaging (MRI) 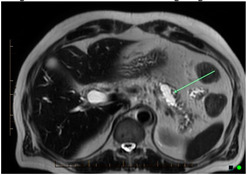	Highest sensitivity and specificity (93% and 89% respectively) Better soft tissue resolution No radiation exposure Better at determining metastasis Better accuracy for assessing local involvement of a pancreatic lesion	Can be difficult to obtain in patients with claustrophobia, metal devises, or allergies to gadolinium (very rare)
